# Resilience against radicalization and extremism in schools: Development of a psychometric scale

**DOI:** 10.3389/fpsyg.2022.980180

**Published:** 2022-11-10

**Authors:** David S. Eldor, Karine Lindholm, Maria H. Chavez, Sander Vassanyi, Michelle O. I. Badiane, Kemal Yaldizli, Petter Frøysa, Christian A. P. Haugestad, Jonas R. Kunst

**Affiliations:** ^1^Department of Psychology, University of Oslo, Oslo, Norway; ^2^Ungdom Mot Vold, Oslo, Norway

**Keywords:** extremism, radicalization, resilience, schools, social equality

## Abstract

Practitioners, policymakers, and researchers alike have argued that the school environment can be both a risk and resilience factor for radicalization and extremism among youth, but little research has tested this directly. Against this background and using a cultural and community psychological approach, we developed a scale to measure resilience against radicalization and extremism in schools focusing on factors that can strengthen social cohesion. A total of 334 school pupils from mostly urban areas in Norway were recruited for this research, of which 233 passed an attention check and were retained for analyses. Participants completed a larger set of items that were derived from theory and the experiences of practitioners and were intended to measure resilience to radicalization and extremism. Exploratory factor analysis identified three dimensions: (1) the perception that the school treats pupils equally no matter their social backgrounds, (2) the perception of the school and its employees as attentive and proactive in meeting pupils’ anger resulting from social and political issues, and (3) the presence of mutual respect. In regression analyses, the equality dimension predicted lower extremist intentions and radicalization as well as four out of five extremism risk factors (i.e., lower anomie, symbolic and realistic threats, and relative deprivation). Mutual respect showed no significant effect, whereas school attentiveness positively predicted three risk factors as well as radicalism intentions and violent intentions, suggesting that such attentiveness may be a response to these issues. In sum, the findings indicate that resilience against extremism in schools may be nurtured especially by creating an egalitarian school environment. Our study provides the first scale assessing such resilience in schools, which can be further tested and developed in future research.

## Introduction

The rising threat of terrorism has attracted the attention of both scholars and governments during the last decades. It has also been a source of public anxiety and political polarization. Thus, identifying social areas which may help prevent radicalization and foster positive intergroup relations on a societal scale represents an urgent issue. It has been proposed that school environments can both reduce and contribute to radicalization and extremism among adolescents. Yet, little research has investigated this directly. Against this backdrop, the present research aimed to develop a scale that reliably assesses resilience to radicalization and extremism in schools.

### Psychological perspectives on extremism among adolescents

Violent extremism has been increasingly studied over the last two decades and the research interest has increased rapidly also within the field of psychology. While the majority of violent extremists are characterized as being young (e.g., Lyall, 2017; Ministry of Justice and Public Security, 2020), comparably few studies have specifically examined radicalization and extremism during youth and adolescence. For long time, psychopathology was seen as a main driver of extremism in this population (see Corner and Gill, 2018, for a general discussion). However, more recently, Harpviken (2020) reviewed the literature and identified five vulnerabilities beyond mental illness, namely “traumatic experiences, early socialization, perceived discrimination, social capital and delinquency” (p. 1; also see Harpviken, 2021). As several of these factors demonstrate, the social context has received increasing attention in recent years. For instance, Lyzhin et al. (2021) likened extremism among youth to socially deviant behavior that results from an identity crisis. Consequently, the authors described extremism as behavior that has the purpose to achieve political change and social equality (also see Nivette et al., 2017). Similarly, Adam-Troian et al. (2021) argue that globalization has heightened different types of threats (i.e., affiliative, economic, existential) that extremism is a response to among youth. Indeed, in a study of high school students, sociopolitical ideology related to social equality was the only significant predictor of pro-violence attitudes (Vukčević Marković et al., 2021).

Whereas we are not aware of theoretical frameworks that specifically have been developed to explain radicalization and extremism in young populations, general perspectives and theories in the field seem applicable. Group relative deprivation – the perception of unjust social and economic difference between the individual’s group and other groups in society – has been proposed as one impactful framework of radicalization (Kunst and Obaidi, 2020). Relative deprivation predicts collective action, including non-normative forms such as violent extremism, which can be viewed as an attempt to change the *status quo* that is perceived as unjust (see Obaidi et al., 2018a; Kunst and Obaidi, 2020). As one grows up, one may become increasingly aware of the comparably low status and potential maltreatment of one’s group at a local and global level. This perception may fuel feelings of relative deprivation and, thus, violent extremism among young people.

Similarly, perceptions of individual- and group-level humiliation and victimization have been found to increase the endorsement of violent extremism and behavioral intentions to engage in violence (Obaidi et al., 2018a), as have experiences of discrimination, exclusion (Obaidi et al., 2019), and marginalization (e.g., see Lyons-Padilla et al., 2015; but see Tahir et al., 2019). A main reason for this observation is that such experiences lead to emotions that have been linked to extremism, most centrally, anger (see Obaidi et al., 2019). Some adolescents and young adults may have grown up with experiences of group-based humiliation, victimization, discrimination, and exclusion, and the resulting anger may drive radicalization and endorsement of violent collective action.

Next, perceptions of symbolic and realistic threats have been linked to radicalization (see Obaidi et al., 2018b). Symbolic threat refers to the perception of a threat to one’s culture, values, or traditions. Realistic threat, meanwhile, refers to perceived threats to aspects such as one’s economic or physical security. Symbolic threat has experimentally been shown to have a causal relationship with endorsement of and intentions to engage in political violence (Obaidi et al., 2018a). Research has further suggested that symbolic threat is more relevant to processes of radicalization and extremism than realistic threat (e.g., Obaidi et al., 2018b). Such threats can also be expected to predict extremism among youth given that adolescence is a critical period of personal and social identity formation.

Finally, another relevant framework of violent extremism is that of significance quest theory (e.g., see Kruglanski et al., 2014). This framework conceptualizes radicalization as a possible pathway to gain or restore the experience of personal or group significance. In line with the theory, the experience of insignificance causally predicts the endorsement of extreme groups and attitudes (Webber et al., 2018). Feelings of insignificance can be operationalized with the concept of anomie (see Travis, 1993). Anomie refers to an experience of lack or degradation of societal cohesion and leadership (Teymoori et al., 2017), involving feelings of alienation, hopelessness, discrimination, and a lack of societal fit (Travis, 1993). For youth as for adults, feelings of anomie may in turn drive radicalization and violent extremism.

### School as an arena to counter radicalization and extremism

Whereas youth and adolescence clearly constitute a phase in which individuals may be especially vulnerable to radicalization or extremism, the social arenas they spend most of their time in may expose them to resilience as well as risk factors. One central social arena for the Norwegian participants in the present study is the school. The Norwegian Education Act stresses that Norwegian pupils have a right, and a *duty*, to primary school education (Ministry of Education and Research, 2019). As an institution with mandatory participation for children and youth, the Norwegian public school is a part of the everyday lives of most Norwegian children, regardless of their backgrounds. The amount of time children and youth spend at school underlines the central role it plays in their lives as an arena of socialization and identity formation. Indeed, the Norwegian Government’s action plan against radicalization and violent extremism emphasizes the importance of school as an arena for developing the democratic values and skills necessary to participate in society and being prepared for work life (Ministry of Justice and Public Security, 2020).

Schools are also arenas where many are exposed to different political attitudes and ideologies than what they may be used to from home. Little surprisingly then, adolescence is a critical time in youth development during which young people develop moral and political ideas about the world (van San et al., 2013). Adolescence can also be a confusing time in which young individuals find themselves between childhood and adult life, which may leave them particularly vulnerable to the involvement in extremist groups (van San et al., 2013; Harpviken, 2020).

As explained by Hardy (2019), a large part of the Norwegian government’s efforts to counter radicalization involves the extensive collaboration between state institutions and non-state practitioners and organizations that work in the field with individuals at risk. The Norwegian youth researchers Vestel and Bakken (2016) emphasize that many young adults who sympathize with extremist views seem to have legitimate concerns but experience that their views are not being taken seriously by authority figures. Their findings indicated that the few participants who did endorse extremist attitudes tended to perceive low social cohesion at school, have poor relationships with other young people, as well as to be dissatisfied with school and educators (Vestel and Bakken, 2016). The authors argued that their findings emphasize that “extremist attitudes should be understood within a framework of concepts such as marginalization and exclusion” (Vestel and Bakken, 2016, p. 135).

The findings of Vestel and Bakken (2016) are in line with those of Swedish researchers who emphasized that pupils’ relationship with teachers have a critical impact on processes that lead to radicalization (Mattsson and Johansson, 2020). Such findings identify the importance of youths’ relationship with adult authority figures, and how authority figures such as school employees (e.g., teachers and social workers) approach youth who sympathize with extremist ideas.

### Proposing a scale to measure resilience to radicalization and extremism in schools

Provided that school is a social arena that may both lead to and prevent radicalization and extremism, measuring its degree of resilience becomes vital. Resilience can be defined as the ability to maintain psychosocial functioning and a coherent sense of self in adverse contexts (see Koirikivi et al., 2021). From a community perspective, it can be understood as a protective factor contributing to positive outcomes and developments when met with significant stressors or potential negative developments, thereby offsetting risk factors and vulnerabilities (Ungar, 2011). Resilience has been proposed as a key factor involved in preventing radicalization and extremism among youth in schools (Weine et al., 2017); however, whereas the facilitation of resources such as physical and emotional safety in school and supportive relationships in the school context have shown to strengthen resilience in youth (Koirikivi et al., 2021) and general scales exist to measure resilience to extremism (e.g., Grossman et al., 2020), resilience scales focusing specifically on schools and the pupil–teacher relationship seem to lack to the best of our knowledge. Thus, although an emerging body of research on extremism has theoretically highlighted the importance of school resilience to counter radicalization amongst youth (e.g., Lösel et al., 2020; Sjøen, 2020), few studies have empirically investigated such resilience. Here, the present research aimed to make a contribution by developing a novel psychometric scale that specifically measures resilience in the domains of radicalization and extremism in schools. Further, with its basis in a cultural and community psychological perspective, the scale aims for a high degree of ecological validity as it is developed together with stakeholders and practitioners.

Based on the existing research on radicalization and violent extremism detailed above and in collaboration with an expert group and the non-profit organization Youth Against Violence (Norwegian: Ungdom mot Vold), we identified various aspects of school resilience that may be of importance at a community, group, and structural level in Norway and potentially other places in the world. Resilience at school may arguably be characterized by an environment where pupils feel treated equally and justly and where all attitudes and identities are recognized. Such an egalitarian and inclusive school environment may counter discrimination and buffer against experiences of relative deprivation, largely through experiences of the equal treatment of pupil. This environment can also be expected to promote feelings of personal significance through the fair treatment and recognition of diverse attitudes and identities.

Next, an aspect of resilience against radicalization in schools may be a safe social environment (thereby countering realistic threat) as well as the presence of tolerance which may be facilitated in parts through open and respectful discussion of diverging values, attitudes, and traditions (thereby countering symbolic threat). As perceptions of humiliation and victimization may increase radicalization by eliciting anger, a resilient school environment may further be characterized by the ability to recognize and accommodate negative emotions displayed by its pupils.

### The Nordic context of extremism

As the present research took a cultural and community psychological approach, it is important to describe the Norwegian context of the study. The arguably most known terror attack committed in the Nordic countries in modern times is the Utøya massacre and Oslo bombing on the 22nd of July, 2011 (Aftenposten, 2021). After setting off a bomb in the Government Quarter in Oslo, killing eight people, the White nationalist Anders Behring Breivik proceeded to a political youth summer camp at Utøya where he shot and killed 69 people, injuring even more. While arguably being the most severe terrorist attack in Scandinavia, many other far-right and Islamist terrorist attacks have been carried out in the last few decades. For instance, in Denmark, there was an Islamist terrorist attack on a synagogue in 2015, killing one person, and injuring several police officers (BBC News, 2015). In Sweden, there was an Islamist terrorist attack on a shopping street in 2017, where five people were killed and several injured (BBC News, 2018). In Norway, a young White nationalist attempted a terrorist attack on a Mosque after having killed his adopted sister who was of Asian origin (Aftenposten, 2019). Most recently, in 2022, an Islamist terrorist attack was conducted on the Pride celebration in Oslo, killing two (Årtun et al., 2022; Yeung et al., 2022). Also of note, Sweden, Denmark, and Norway (in this order) were among the Western countries with the highest number of ISIS foreign fighters both per capita and per Muslim population between 2012 and 2016 (Benmelech and Klor, 2016).

In 2020, the Norwegian government launched an updated version of the action plan against radicalization and extremism (Ministry of Justice and Public Security, 2020). It contains two reports concerning the demographics of individuals associated with radical Islamist and far-right extremist communities in Norway. For both ideologies, the individuals are characterized as being young men, primarily under the age of 30. This observation further emphasizes the need to understand radicalization among young people in this Nordic setting.

### The present research

Against the review of the literature and meetings with relevant practitioners and experts, the present research aimed to develop a quantitative scale of community resilience against radicalization and extremism in schools (RARES). A large item pool was initially created based on the literature and the experience of the practitioners (see the section “Materials and methods” for details). This pool was then administered together with validation measures among secondary school pupils in Norway. The validation measures included an existing measure of general school resilience that was expected to correlate positively with the new scale, providing convergent validity. Next, it included criterion validity measures (of relative deprivation, symbolic and realistic threat, collective anger, and anomie), that were expected to be negatively associated with the new resilience scale. Third, the survey included two predictive validity measures (of radicalization intentions and violent behavioral intentions) that were expected to be negatively predicted by the new scale.

The present research has both theoretical and practical implications. Theoretically, it extends knowledge about the role of resilience in the domains of extremism and radicalization. Whereas at least one scale exists to assess general resilience against extremism among youth (Grossman et al., 2020), we are unaware of extremism resilience scales specifically focusing on the school context and the quality of pupil–teacher relationships. As the reviewed literature suggests, school is a critical social arena that may both lead to and hinder the development of extremism and radicalization in young people. Thus, empirical insights into the resilience against extremism in this social arena may have important implications for theoretical frameworks of both resilience and radicalization, impacting future research. Practically, creating a measure of RARES and testing its relationships to various extremism-related outcomes can help inform school policies and the work of non-governmental organizations and practitioners. A psychometric measure will also allow schools to assess their own resilience and can be used as an endpoint in interventions.

To understand the cultural and community psychological approach of this research and to ensure transparency, it is important to give a brief overview of the organization that the researchers collaborated with and its involvement. The organization Youth Against Violence contributed with their expertise by highlighting the research gaps based on their own practical experience and knowledge of the field, as well as by providing theoretical, practical, and contextual knowledge. Youth Against Violence played a critical role in gathering the data, both through establishing contacts with the schools in which the participants for this study were recruited, as well as the hands-on data collection through the administrations of the questionnaire.

## Materials and methods

### Participants

A total of 334 participants were recruited for this study. Of these, 101 failed an attention check that asked them to select a specific response on a Likert scale presented as part of the resilience items, leaving a sample of 233. This sample was close to common recommendations for factor analyses (e.g., a ratio of 5 participants per variable in analyses with at least 200 participants; Mundfrom et al., 2005). In this sample, 58.5% were female (*n* = 136), 32.2% were male (*n* = 75), 2.1% (*n* = 5) identified as “other,” and 3.4% (*n* = 8) did not answer this question. All participants were over 16 years old. To ensure anonymity, the exact age of participants was not recorded. The participants were attending either secondary school (Norwegian: Ungdomsskole) or upper secondary school (Norwegian: Videregående skole) in mostly urban areas of the southeastern part of Norway. Of the total sample, 26.2% reported that one or both of their parents had a non-European ethnic background, while 69.5% reported that neither of their parents had a non-European ethnic background.

### Procedure

The present study was approved by the Internal Review Board of the Department of Psychology at the University of Oslo (Nr. 19699599) and the data were collected online through either a link or a QR code. The survey included 86 items, taking approximately 15 min to complete. The participants were invited to take part in a study that was described as investigating the school environment and political attitudes in society, developed in cooperation with Youth Against Violence. Information about the study, voluntary participation, and confidentiality were presented in the informed consent form.

The recruitment was conducted through targeted sampling, specifically focusing on pupils attending upper secondary school in the southeastern part of Norway. The survey was distributed to the respondents by either the authors, Youth Against Violence, teachers, or the school administrations. The study consisted of several statements where the participants were asked to indicate how much they agreed or disagreed on a Likert scale ranging from 1 (*completely disagree*) to 7 (*completely agree*), with 4 as a neutral option (*neither agree nor disagree*). The participants could choose not to answer any question except for the informed consent where the participants confirmed that they were above 16 years of age and that they wanted to take part in the study. At the end of the survey, participants were asked demographic questions regarding their gender and their parents’ ethnic background. The participants were provided with contact information for questions regarding the study and were, following the completion of the survey, thanked for their participation.

### Measures

The study consisted of nine measures, of which eight were already established measures used in previous research. All the established measures were forward-back translated from English into Norwegian. All measured instruments are reported in the present manuscript.

#### Resilience against radicalization and extremism in schools scale

The construction of the scale followed the steps that typically are recommended and were summarized by, for instance, Boateng et al. (2018) or Carpenter (2018; see Clemmow et al., 2022, for the application of the steps in the field of extremism research). First, resilience to extremism and radicalization at school was specified as the domain of interest. A literature review suggested that there were no existing measures. Thus, an item pool was created considering the established risk factors of extremism and radicalization reviewed in the introduction and based on the experiences of the expert group.

All items dealt specifically with the school environment’s handling of and interaction with the pupils’ political and societal diversity and frustrations. First, seven items assessed *emotional relief*. These items sought to measure the degree to which employees and significant persons in the school environment are available for conversation, listening, and emotional venting for the pupil (e.g., “When I feel angry over things happening in the world, I have employees at school whom I can talk to”). The items refer to societal, political, and unjust dilemmas in the world, and are aimed to counter both feelings of injustice, and risk factors linked to symbolic and realistic threat. Next, *recognition* items assessed how serious and/or sincerely the pupils felt that the employees addressed their concerns and respected their opinions (e.g., “I can express my opinions to the school employees even if they disagree with me”). *Sensitivity and vigilance* items tapped into the degree to which the pupils perceived the school employees to be sensitive to their frustrations and attempted to help (e.g., “The employees at school notice it and try to help when I am angry over unfair things in the world”). *Intervention from employees* items measured the degree to which the pupils perceived that school employees facilitate discussions among the pupils regarding “strong opinions” (e.g., “When someone at school expresses strong opinions, it is brought into a discussion where all sides are heard,” or, “At school, we have discussions where I get to talk about values that are important to me”).

*Need for meaning* items measured the perceived assistance from school employees regarding the pupils’ feeling of meaningfulness and sense of place in society (e.g., “If I had felt that my life had no direction, the employees at school would try to help me”). *Need for belonging* items measured the pupils’ experience of fitting in, inclusion, and acceptance of alternative opinions, as well as school employees’ ability to strengthen these factors (e.g., “At my school, we make an effort to create an inclusive school environment”). *Justice* items tapped into the perceived ability of school employees to treat pupils equally and fair (e.g., “The school employees make me feel as important as most other pupils”). Finally, *tolerance* items measured the perceived acceptance of divergent opinions, different backgrounds, and respect regardless of one’s cultural belonging (e.g., “At our school, it is important that pupils are respected regardless of their background”). Thus, all items were aimed to counter the proposed risk factors relative deprivation, symbolic and realistic threat, collective anger, and anomie. All exact item wordings can be found in the Supplementary materials, forward-back translated into English.

The initial item pool was evaluated by the expert group and evaluated and pre-tested by representatives of the target population (i.e., a group of pupils) to ensure their comprehensibility and ecological validity. Changes were made to the items when needed. This resulted in changes in about 40% of the items, but no item was deleted. The final set of items was then administered to the study sample. Based on exploratory factor analyses, items without cross-loadings were retained and the reliability of each dimension was evaluated. Finally, tests of construct validity were conducted with the various measures that were included for this purpose.

#### Established scales

##### Convergent validity

###### Safety and connectedness

A measure composed of five items (e.g., “I feel safe in my school”; α = 0.90) was adopted from Hanson and Voight (2014) and investigated the pupils’ perception of feeling safe and connected within their school environment.

##### Criterion validity

###### Relative deprivation

Six items (e.g., “Members of my ethnic group should have the same opportunities to improve their lives as everyone else in Norway”; α = 0.82) adapted from Obaidi et al. (2019) investigated the pupils’ perception of relative deprivation.

###### Symbolic threat

Three items (e.g., “My ethnic culture is threatened by other groups in Norway”; α = 0.91) adapted from Obaidi et al. (2018b) investigated whether the pupils experienced symbolic threat.

###### Realistic threat

Three items (e.g., “My ethnic group is unsafe due to other ethnic groups in Norway”; α = 0.95) adapted from Obaidi et al. (2018b) investigated the pupils’ perceived realistic threat.

###### Collective anger

Three items (e.g., “I feel angry when I think of injustices committed against my ethnic group”; α = 0.95) adapted from Obaidi et al. (2019) investigated collective anger.

###### Anomie

Seven items (e.g., “My whole world feels like it’s falling apart”; α = 0.85) adopted from the MOS Alienation scale of Travis (1993) investigated the pupils’ feeling of anomie.

##### Predictive validity

###### Radical intentions scale

We adopted a scale from Moskalenko and McCauley (2009), comprised of four items (e.g., “I would participate in a public protest against oppression of my ethnic group even if I thought the protest might turn violent”; α = 0.83). The measure investigated the pupils’ willingness to participate in radical activities in society.

###### Violent intentions

We adapted a measure from Obaidi et al. (2018b). The scale consisted of seven items (e.g., “As a last resort I’m personally ready to use violence for the sake of my ethnic group.”; α = 0.90).

### Analysis

Before running the analyses, Little’s MCAR test was conducted to see if there was a pattern in missing values. We then conducted an exploratory factor analysis with oblique rotation on the items that were developed to measure resilience to radicalization and extremism in schools. Based on the results, we created a scale represented by three different dimensions. Next, we ran group comparisons to test for gender and ethnic differences on the resilience dimensions. For validation, we then investigated the correlations between the resilience scale that we developed and the established validation measures. Linear regression analyses with the three scale dimensions and established resilience measure (Hanson and Voight, 2014) as predictors were conducted to examine their unique contributions. The statistical analyses were conducted with IBM SPSS statistics, version 27.0 (IBM Corp, 2020).

## Results

Little’s MCAR was significant (*p* < 0.001). Results indicated that several participants had skipped the measures of radicalism intentions and violent behavioral intentions. Thus, missing values were not imputed, and cases were excluded listwise in analyses. An exploratory factor analysis with maximum likelihood estimation was conducted with the resilience items. Bartlett’s test of sphericity was significant (*p* < 0.001) and the Kaiser–Meyer–Olkin measure of sampling adequacy was 0.949, well above the common cut-off of 0.6. Based on the scree plot (see Supplementary materials), three components were retained, explaining 53.02% of the total variance in the model. The first factor accounted for 42.75% of the explained variance (eigenvalue = 20.52), the second factor accounted for 6% (eigenvalue = 2.88) and the third factor accounted for 4.27% (eigenvalue = 2.05). Oblique rotation was used as the factors could be assumed to correlate. The first factor was named “school attentiveness” as the common denominator for the items was the school employees’ attentiveness toward anger among the pupil and efforts to help them find a meaning and direction in life (e.g., “The employees at school notice if I am angry due to something happening in the world” or “If I would feel that my life had no direction, the employees at school would try to help me out”; see Table 1). The second factor was named “equality,” as the items all related to equal treatment and acceptance (e.g., “I feel that the school employees treat me the same as other pupils”). The third factor was composed of three items representing the experience of “mutual respect” at school (e.g., “At school, everyone is respected regardless of which group they belong to in society”).

**TABLE 1:** Results from exploratory factor analysis with oblique rotation.

	Factor		
	
	Attentiveness	Equality	Respect
The employees at school notice if I am angry due to something happening in the world.	**0.860**	0.274	0.106
When I am angry because of unfair things happening in the world, a school employee will notice it and try to help me.	**0.793**	–0.020	–0.058
When I am angry due to injustices in the world, there are school employees who ask if I am alright.	**0.774**	–0.008	–0.034
The employees at school notice it and try to help when I am angry over unfair things in the world.	**0.731**	–0.043	–0.028
The employees see it when I am frustrated over events in the world.	**0.727**	0.019	–0.007
When I feel angry over things happening in the world, I have school employees that I can talk to.	**0.694**	–0.187	–0.035
It is easy for me to talk to employees at school when I feel anger over political issues.	**0.693**	0.012	0.023
The school employees are easy to find when I need someone to talk to about injustices in the world.	**0.660**	–0.017	0.212
When someone at school expresses strong opinions, it is brought into a discussion where all sides are heard.	**0.642**	0.003	0.182
I can share frustrations I feel due to injustices in the world with school employees.	0.618	–0.338	–0.140
If I had had difficulties understanding the meaning with my life, the school employees would try to help me.	**0.590**	–0.112	0.178
When I am angry over things happening in the world, I have employees at school who take me seriously.	**0.576**	–0.291	0.049
At my school, the employees help us discuss strong political opinions in an open manner.	**0.561**	–0.169	0.003
The employees at school help create a good discussion when a pupil at the school expresses political opinions that makes others react.	**0.544**	–0.169	0.153
If I would feel that my life had no direction, the employees at the school would try to help me out.	**0.539**	–0.297	0.079
The school employees would help me if I was unsure of my place in society.	**0.532**	–0.264	0.118
The school employees help me find my role in society if I need it.	**0.522**	–0.289	0.023
When I feel insecure concerning my future, the school employees try to help me.	**0.514**	–0.188	0.081
When someone at my school shows opinions that others think are hurtful, it is dealt with in a good way by the school employees.	0.514	–0.001	0.436
At school, we have discussions where I get to talk about values that are important to me.	**0.497**	–0.052	0.129
None of the school employees notice it if I am frustrated over things happening in the world.	−**0.477**	–0.123	0.036
The school employees work toward tolerance of people with different backgrounds, political views, ethnicities, religions, and orientations.	0.452	–0.125	0.321
If I am angry because of injustices in the world, there is someone at school I can share it with.	0.356	–0.281	0.016
None of the school employees would try to help me if I was unsure of the meaning of my life.	–0.344	0.149	0.000
It is difficult to get contact with the school employees when I am angry over things happening in the world.	–0.307	0.079	0.121
I feel that the school employees rarely take what I care about in the world seriously.	–0.260	0.086	–0.048
I feel that the school employees treat me the same as other pupils.	0.000	−**0.803**	0.075
I feel that the school employees treat me unfairly.	0.138	**0.735**	–0.010
I feel that the school employees give me the same opportunities as the other pupils.	0.045	−**0.717**	0.028
The employees treat me as fairly as they treat most other pupils at school.	0.050	−**0.668**	0.149
The employees of the school work as much as they can for me to succeed as they work for the other pupils.	0.164	−**0.640**	0.105
I feel accepted at the school even if I might have different opinions than the others.	0.051	−**0.596**	0.190
The school employees make me feel as important as most other pupils.	0.242	−**0.547**	0.109
I feel that I, with the opinions that I have, am included at the school.	0.103	−**0.535**	0.183
I feel that my opinions are heard and respected even if the school employees disagree with me.	0.303	−**0.510**	0.132
I feel like a part of the school’s community even if my opinions can be different from the others.’	0.163	−**0.468**	0.244
The school employees take my opinions seriously even if they disagree with me.	0.384	–0.455	0.048
My opinions are respected by the school employees even if they might disagree with me.	0.290	−**0.445**	0.162
At my school we make an effort for there to be an inclusive school environment.	0.269	−**0.444**	0.190
At our school it is important that pupils are respected regardless of their background.	0.113	−**0.441**	0.160
I can tell my opinions to the school employees even if the disagree with me.	0.333	–0.423	0.180
I feel that I fit in at school even if I have some opinions that are different from those of others.	0.163	–0.377	0.336
At school, we rarely talk about how to behave with people who are different.	–0.075	0.131	0.068
At school, everyone is respected regardless of which group they belong to in society.	0.060	–0.128	**0.750**
At school, everyone is respected as they are.	0.158	–0.060	**0.710**
I experience that the pupil environment at the school accepts people who are different.	0.068	–0.238	**0.486**
I feel that those who have different opinions than most others are excluded from the school environment.	–0.092	–0.038	–0.321
At school, there is little acceptance of pupils who stand out as different in the society.	0.112	0.114	–0.274

Extraction method: Maximum Likelihood. Rotation method: Oblimin with Kaiser Normalization. Items in bold have loadings >0.4 on their main factor and cross-loadings below 0.32. These items were used to create the scales.

**FIGURE 1:**
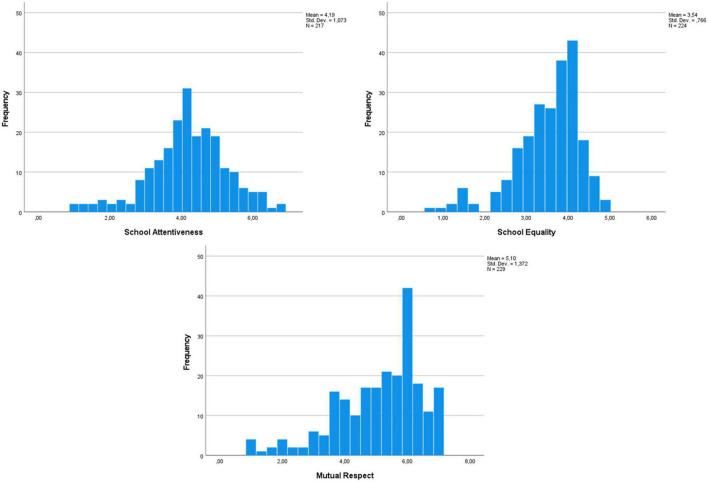
Response distributions of the resilience scales.

For consistency, items that clearly loaded on one factor (>0.4) and showed cross-loading below ± 0.32 on the other two factors were selected when computing the scales (see Tabachnick and Fidell, 2001). Thus, we ended up with 19 items on school attentiveness (α = 0.94), 13 items on equality (α = 0.94), and three items for the respect scale (α = 0.83). The response distributions on the scales can be seen in Figure 1. Next, we ran independent sample *t*-tests to investigate whether gender or ethnic differences were observed for the two resilience subscales. No significant gender difference was observed for school attentiveness, *t*(199) = 0.61, *p* = 0.537, equality, *t*(117.29) = −1.72, *p* = 0.088, or mutual respect, *t*(205) = −1.02, *p* = 0.307. A significant difference was observed between ethnic majority and minority members for school attentiveness, *t*(209) = −2.31, *p* = 0.028, but not for equality, *t*(214) = 0.08, *p* = 0.934, or mutual respect, *t*(217) = −1.31, *p* = 0.193. Minority members reported higher school attentiveness (*M* = 4.45, *SD* = 1.18) than majority members (*M* = 4.08, *SD* = 1.02).

We then estimated the correlations between the subscales and the established measure (see Table 2). All three scales were positively correlated with the existing school resilience scale (i.e., the safety and connectedness scale by Hanson and Voight, 2014), providing convergent validity. In terms of risk factors, school attentiveness negatively correlated with anomie. Equality negatively correlated with symbolic threat, realistic threat, and anomie. Mutual respect negatively correlated with anomie, symbolic threat, and realistic threat. This provided criterion validity for the subscales but especially so for the equality and respect subscales. Equality was negatively correlated with radicalism intentions and violent behavioral intentions, whereas mutual respect was negatively correlated with violent behavioral intentions. This provided support for the predictive validity of two of the three sub-scales.

**TABLE 2:** Correlations between main study variables.

	2	3	4	5	6	7	8	9	10	11
1. Attentiveness	0.73***	0.54***	0.53***	−0.45***	–0.06	–0.01	0.08	0.03	0.04	–0.00
2. Equality		0.70***	0.68***	−0.63***	−0.25***	−0.24***	–0.07	–0.08	−0.15*	−0.26***
3. Respect			0.47***	−0.49***	−0.14*	−0.18**	–0.08	0.01	–0.07	−0.20**
4. Safety and connectedness				−0.50***	−0.24***	−0.21**	–0.07	–0.03	–0.05	−0.18**
5. Anomie					0.28***	0.29***	0.22**	0.19**	0.24***	0.34***
6. Symbolic threat						0.69***	0.46***	0.58***	0.41***	0.28***
7. Realistic threat							0.54***	0.55***	0.35***	0.38***
8. Anger								0.55***	0.42***	0.29***
9. Relative deprivation									0.48***	0.28***
10. Radicalism intentions										0.561***
11. Violent intentions										

***Correlation is significant at <0.001 level (2-tailed). **Correlation is significant at the 0.01 level (2-tailed). *Correlation is significant at the 0.05 level (2-tailed).

To estimate the unique contributions of the four resilience scales (equality, school attentiveness, mutual respect, and safety & connectedness), we estimated linear regression models with each of the risk factors and the radicalism and violent intentions scales as dependent variable. As displayed in Table 3, the equality resilience variable had the most explanatory power. It predicted lower levels on all variables except for anger. School attentiveness unexpectedly predicted *more* symbolic threat, realistic threat, anger, radicalism intentions and violent behavioral intentions. As most of these association were non-significant and approached zero in the correlation analyses (Table 2), they reflect suppressor effects that need to be interpreted with cation. The safety and connectedness scale only predicted lower scores on the anomie variable. Mutual respect predicted none of the variables significantly.

**TABLE 3:** Linear regression analyses with equality, attentiveness, mutual respect, and safety and connectedness as predictors.

Dependent variables	Attentiveness			Equality			Mutual respect			Safety and connectedness			*F*	*R* ^2^
						
	*B*	β	*p*	*B*	β	*p*	*B*	β	*p*	*B*	β	*p*		
Anomie	–0.01	–0.01	0.892	−**0.78**	−**0.44**	<**0.001**	–0.09	–0.08	0.260	−**0.17**	−**0.16**	**0.033**	33.21***	0.39
Symbolic threat	**0.28**	**0.21**	**0.040**	−**0.58**	−**0.30**	**0.022**	0.07	0.06	0.497	–0.18	–0.17	0.082	4.70**	0.09
Realistic threat	**0.46**	**0.32**	**0.001**	−**0.68**	−**0.34**	**0.010**	–0.04	–0.04	0.689	–0.14	–0.12	0.191	5.63***	0.10
Anger	**0.36**	**0.22**	**0.031**	–0.37	–0.16	0.239	–0.07	–0.06	0.552	–0.05	–0.04	0.719	1.41	0.03
Relative deprivation	0.12	0.11	0.306	−**0.46**	−**0.28**	**0.038**	0.14	0.15	0.109	0.07	0.07	0.461	1.29	0.03
Radicalism intentions	**0.30**	**0.24**	**0.019**	−**0.80**	−**0.44**	**0.001**	0.09	0.09	0.349	0.12	0.11	0.231	3.00*	0.06
Violent behavioral intentions	**0.51**	**0.38**	<**0.001**	−**0.93**	−**0.49**	<**0.001**	–0.05	–0.04	0.638	0.00	0.00	0.984	7.10***	0.12

***Correlation is significant at <0.001 level (2-tailed). **Correlation is significant at the 0.01 level (2-tailed). *Correlation is significant at the 0.05 level (2-tailed). Significant effects are presented in bold.

## Discussion

School has been put forward as important arena of youth socialization and development during a critical and vulnerable life period. Unsurprisingly, it has been described as having a critical role in processes of radicalization and extremism, but this has rarely been tested directly. In the present research, we took a community psychological approach, assessing RARES. The results identified three factors: school attentiveness, equality, and mutual respect. School attentiveness captures the extent to which the pupils perceive that school employees are attentive to them and their emotional wellbeing, particularly concerning frustrations relating to societal and political issues. This factor can, thus, be described as a form of social support that is directed toward emotional experiences that are known to be central to extremism and the development of it. The second factor – equality – captures the extent to which the pupils perceive fair treatment by the school employees and the school environment to be inclusive and egalitarian. The third factor – mutual respect – dealt with the degree to which the pupils felt that people regardless of their backgrounds were respected at school. Of the three factors, equality clearly seemed to have the most important resilience function when it comes to extremism and radicalization.

Various steps were taken to validate our scales. First, the study examined their convergent validity via an already established measure of school resilience. All three scales correlated significantly and positively with the safety and connectedness measure of Hanson and Voight (2014). This suggests that the scales do provide valid measures of school resilience. In terms of criterion validity, equality was significantly and negatively correlated with realistic threat, symbolic threat, and anomie in terms of zero-order correlations. In regression analyses, it negatively predicted these risk factors and also relative deprivation. The fact that equality was linked to lower experiences of realistic threat, symbolic threat, and relative deprivation may be interpreted as reflecting the absence of intergroup conflict, disunity, grievances, and threat. Instead, pupils may perceive fair treatment irrespective of their backgrounds or group memberships. The negative relationship between equality and anomie may be more complex. The items of the anomie measure differ qualitatively, involving aspects of alienation, hopelessness, perceived discrimination, and societal misfit. Together they provide a formative measure of anomie defined as a general sense of perceived societal and leadership unraveling (Teymoori et al., 2016). Whereas this study cannot investigate the causal relationship between equality and anomie, it could be argued that the experience of equality in the school context may increase the experience of general social cohesion.

School attentiveness was significantly and negatively related to anomie. Research by Vestel and Bakken (2016) found that extremist attitudes among youth in Norway were correlated with aspects related to anomie (e.g., poor relationships to peers and dissatisfaction with and mistrust of the school and educators). Our results conceptually replicate this finding and emphasize the link between high school attentiveness to the anger and frustrations of their pupils and low feelings of anomie. Indeed, the sizable, negative associations with anomie highlight that one key process through which equality and school attentiveness may buffer against extremism is by fostering a sense of social cohesion. However, unexpectedly, school attentiveness was *positively* related to symbolic threat, realistic threat, and anger when controlling for the effects of the other scales. These effects likely represent suppressor effects and need to be interpreted with caution. They may suggest that once the other factors are controlled for, a higher degree of school attentiveness may reflect a *response to* the presence of problematic issues and collective emotions (i.e., anger) in the school environment.

Whereas the mutual respect scale was related to lower levels of symbolic and realistic threats and anomie in terms of zero-order correlations, it did not predict any risk factor in the regression analyses. This finding suggests that mutual respect may have little benefits beyond the effects of equality. However, it is important to note that the scale was made up by only three items. Thus, its measurement may have been too narrow to fully encompass all facets of the construct.

Neither mutual respect nor equality were significantly related to relative deprivation or collective anger in zero-order correlations, and neither variable predicted anger in the regression analyses. The items measuring relative deprivation and collective anger largely involved an upward comparison from the perspective of an underprivileged or threatened ethnic group. As most of our participants were ethnic majority-group members, it is possible that the measure was not experienced as relevant to them (but see Obaidi et al., 2021). This low ecological validity may explain the absence of significant relationships with our resilience measures.

In terms of predictive validity, equality was found to be significantly negatively associated with violent behavioral intentions and radicalism intentions, both in terms of zero-order correlations and regressions. In an egalitarian school context, pupils may be less likely to experience power asymmetries, which otherwise increase violent intentions aimed at restoring the fair treatment of oneself or one’s group or ensuring one’s groups dominant position (Kunst and Obaidi, 2020). It is possible that equality may relate to lower extremism through the absence of experiences of injustice and discrimination, two factors which may increase radicalization (e.g., see Obaidi et al., 2018a; Charkawi et al., 2020). Pupils who experience that their emotional states (especially with respect to societal and political conditions) are recognized by the school employees may feel less alienated and marginalized and experience more self-worth and a more grounded self-identity (e.g., see Hogg and Adelman, 2013; Hogg et al., 2013).

Regression analyses showed that the equality scale was superior to the three other scales in predicting risk factors and extremist outcomes. Thus, equality may have a central role in resilience against and prevention of radicalization and extremism, perhaps especially among youth. This finding supports earlier research showing that inequality predicts extremist outcomes, potentially because it shifts ideological responses to it (Kunst et al., 2017). Thus, our results may suggest that pupils direct and personal experiences with inequality at school generally is most predictive of their political attitudes and intentions. The fact that the attentiveness scale was positively related to several outcomes may make sense from a theoretical perspective. If a school environment is characterized by problematic issues such as the presence of threats and anger, school attentiveness should naturally be higher. However, then the attentiveness scale may be less suited to detect resilience in schools.

Although our correlational data did not allow us to test causal mediation models, the results may suggest that school resilience against radicalization and extremism may be both buffering as well as directly protective. For instance, the equality scale was negatively related to many risk factors, whereas it also negatively predicted violent behavioral intentions and radicalization.

Interestingly, ethnic minority members scored significantly higher on the school attentiveness measure, whereas no significant ethnic differences were observed on the equality or mutual respect measures. Given their lower status in society and their membership in groups that often are suffering from oppression internationally, minority pupil may have experienced grievances regarding the treatment of their group more often than majority pupil (Obaidi et al., 2018a). As such, teachers and other staff may have attended to them more frequently. It may also be that school employees generally are more sensitive to minority students’ concerns for these reasons. The fact that no significant differences were observed on the equality or mutual respect measures is uplifting as it may suggest the absence of ethnic discrimination. However, it is important to note that we, due to privacy concerns, did not assess the pupil’s specific ethnic group membership. As such, white immigrants (i.e., Poles) would be grouped together with non-Western groups that generally experience more discrimination, which may have camouflaged some differences.

Our study was administered in Norwegian schools located in regions close to the capital. It is important to take the respective context into consideration when studying resilience (see, e.g., Theron et al., 2022). Several factors present in the Norwegian context likely had an impact on the findings of this study. Norway has seen three major terrorist attacks over the past two decades, each likely committed by lone-actor extremists without the support of any organized group. This is in line with findings such as those by Vestel and Bakken (2016), who observed that the few participants that did endorse extremist attitudes were more likely to report alienation or poor social relations to other peers. Thus, it is possible that, for instance, the anomie factor plays a particular role in the Norwegian context, whereas other factors may be more important in different contexts.

In general, Norway tends to be characterized as a highly functioning and resourceful welfare society (Meuleman et al., 2018). Its population scores high on support for social rights and egalitarianism, while simultaneously tending to endorse more severe retributions for individuals who are perceived to take advantage of the system (Schwartz, 2006; Meuleman et al., 2018). In support of this, Norway has a GINI coefficient (i.e., a measure of income inequality) of 0.26, which is very low in international comparison and has remained relatively stable over the past decade (Meuleman et al., 2018; Statistisk Sentralbyrå, 2022). Thus, the strong emphasis on egalitarianism in Norway may explain why this factor was most consistently linked to extremism outcomes and risk factors in the present study.

### Limitations, practical implications, and future directions

The findings of the current study must be interpreted with awareness of certain weaknesses. Efforts were made to recruit a sample of participants that reflected the demographics of the target population, however, the sample ended up with a too low representation of participants with an ethnic minority background. Thus, the sample consisted primarily of so-called WEIRD participants (Henrich et al., 2010). Further, around one third of the participants did not pass the attention check and values were found not to be missing at random. Nevertheless, it has to be noted that the rate of failed attention checks in the present study are not unusual in high school pupil samples (see e.g., Evangelou et al., 2008; Saleem et al., 2022; also see Hauser and Schwarz, 2016).

Firstly, it should be mentioned that our total item pool for the resilience scale consisted of a high number of items, which may have contributed to response fatigue and inattentiveness. Secondly, many of the questions dealt with sensitive topics and may elicit a response bias. For instance, some participants skipped the radicalism and violent behavioral intentions scales. Pupils with a minority background, especially young Muslims, may have perceived the statements as stigmatizing, although no negative reactions were reported during the data collection. Negative reporting on immigration and the systemic use of cultural stereotypes is not uncommon in Norwegian mainstream media discourses (Wiggen, 2012), and is common in alternative media, especially following terrorist attacks (Wold, 2022). Muslim youth experience widespread forms of anti-Muslim hostility and discrimination, especially relating to Islamist terror organizations, but have also been found to actively challenge such prejudice with various forms of everyday resistance strategies (Banafsheh et al., 2021). It is thus possible that some participants in the present study decided not to respond as a protest strategy to oppose such perceived stereotyping.

It is also important to note that social desirability may have influenced scores on our measures. Given their sensitive content and the strong societal norms condemning violent collective action, participants may have shown a response bias when answering statements, especially those related to extremist intentions. Moreover, even though the study was anonymous, they may have feared that their responses would be shared with the teachers or other staff. Related to this, it must be noted that our radicalization and extremism measures were very skewed. Such a distribution is expected in most populations and samples as most people per definition do not have radical or violent intentions nor hold extreme attitudes (e.g., see Koirikivi et al., 2021; Jungkunz, 2022). Nevertheless, the limited variance in the measures may have made it difficult to detect significant correlations with some of the resilience scales.

Our study did not measure all variables that can be seen as relevant in the school context, such as treatment by pupils and peer relationships, nor did it measure relevant variables outside of school such as negative social experiences including discrimination or a lack of parental support as discussed by Harpviken (2020). As we noted earlier, it is also important that our study was correlational and thus cannot assess causal relationships. Many studies have, however, established the causal role of resilience in a variety of domains (e.g., see Herrman et al., 2011). Still, additional research is needed to investigate the causal relationship between our resilience measures and extremism variables. Interventions and longitudinal research may offer important insights in this respect.

We emphasize that the present study should be seen as the first step in developing a scale to measure resilience to extremism and radicalization in schools. Only about 50% of the variance was explained by the retained factors, suggesting that the selected items can be further improved. Moreover, a next vital step is to confirm the scales’ factor solution in another sample. Future studies may also profitably test for the measurement invariance between key demographic categories (e.g., gender, ethnicity) with samples that provide sufficient statistical power. Further, in the present study, we included a large number of items when computing the scale to maximize the breadth of the constructs. For practical reasons, it may be meaningful to select the items with the highest factor loadings in future research, thereby testing short forms of the present scales.

Lastly, it is important to mention that the questionnaire developed for the present study measured intentions and not actual extremist behavior. Whereas this limitation is due to ethical reasons that are common within extremism research, it is important to note that the intentions we assessed do not necessarily translate fully into behavior.

Our findings have theoretical implications and may inform guidelines for preventing radicalization and extremism in schools through the creation of social cohesion. The present research thus corresponds closely to the call of the “‘Divided or united’: Strengthening Social Cohesion for Well-being and Prosperity” special issue. By developing a scale to measure resilience to extremism in schools and identifying one potentially impactful dimension (i.e., equality), it provides information on how social cohesion can be build and strengthened within society via social institutions. The results primarily highlight the beneficial roles that school employees can play by showing vigilance and promoting equality. As radicalization tends to onset at a fairly young age (e.g., Lyall, 2017; Ministry of Justice and Public Security, 2020), our scale may be valuable in testing the effectiveness of interventions or to generally allow a school to assess its levels of resilience. Yet, future research with representative samples is needed to establish the average level of resilience in schools in a certain country (i.e., population means) to allow for the detection of deviation from this average.

Future research should also investigate whether resilience functions differently for extremism related to different ideologies. While sharing many characteristics (e.g., see Hogg and Adelman, 2013; van Proojien and Kuijper, 2020), it is possible that extremism related to different ideologies and organizations shows different correlations with our resilience scales. Moreover, it would be interesting to test whether our scale predicts attitudes and intentions concerning lone-actor and group-based radicalization and extremism differently (e.g., see Gill et al., 2014; McCauley and Moskalenko, 2014; Jasko et al., 2020).

## Conclusion

The present research developed a scale to assess school resilience to radicalization and extremism. Three factors were identified, namely equality, school attentiveness, and mutual respect. Especially equality, reflected in the perceived equal treatment of all pupils regardless of their backgrounds and an inclusive environment, was related to less extremism, including a series of risk factors. Thus, to bolster a school’s resilience, teachers and other staff should put emphasis on creating egalitarian norms and environments as well as being proactive in meeting pupils’ political anger.

## Data availability statement

The original contributions presented in this study are included in the article/Supplementary material, further inquiries can be directed to the corresponding author.

## Ethics statement

The studies involving human participants were reviewed and approved by Internal Review Board, Department of Psychology, University of Oslo. Written informed consent from the participants’ legal guardian/next of kin was not required to participate in this study in accordance with the national legislation and the institutional requirements.

## Author contributions

DE, KL, MC, SV, MB, CH, and JK designed the study and wrote the main text. DE, KL, MC, SV, MB, KY, and PF collected the data. DE, KL, SV, and JK analyzed the results. CH, KY, and PF provided feedback. All authors contributed to the article and approved the submitted version.
