# Supplementary material for: Resilience against radicalization and extremism in schools: Development of a psychometric scale
Source: Front Psychol. 2022 Nov 10;13:980180. doi: 10.3389/fpsyg.2022.980180 (PMC9685524; doi:10.3389/fpsyg.2022.980180)
Supplement: Supplementary file 2 [file Table_2.DOCX]

***Supplementary Material***


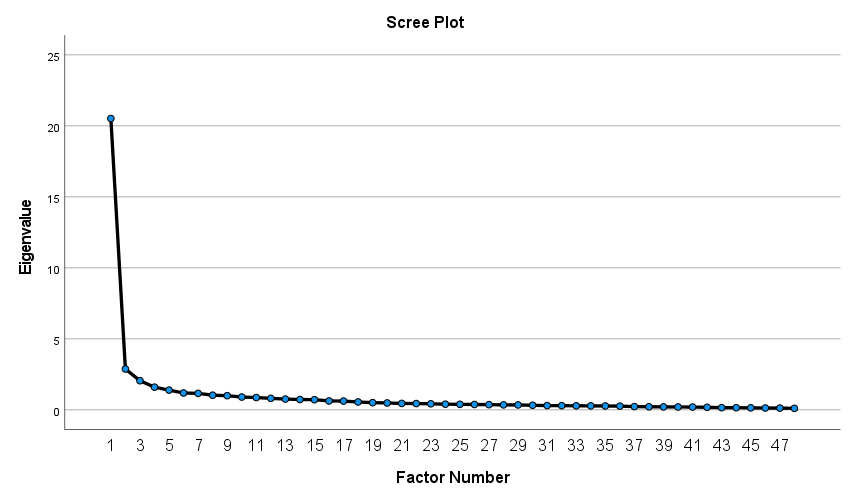


**Supplementary Figure 1.** Scree plot from exploratory factor analysis.

| **Supplementary Table 1** | | | |
| --- | --- | --- | --- |
| *Eigenvalues, Percentages of Variance, and Cumulative Percentages in Exploratory Factor Analysis* | | | |
| Factor | Eigenvalue | % of variance | Cumulative % |
| 1 | 20.519 | 42.747 | 42.747 |
| 2 | 2.878 | 5.995 | 48.743 |
| 3 | 2.051 | 4.273 | 53.016 |
| 4 | 1.599 | 3.330 | 56.346 |
| 5 | 1.391 | 2.899 | 59.245 |
| 6 | 1.187 | 2.474 | 61.719 |
| 7 | 1.162 | 2.420 | 64.138 |
| 8 | 1.032 | 2.151 | 66.289 |
| 9 | .998 | 2.080 | 68.369 |
| 10 | .902 | 1.879 | 70.248 |
| 11 | .866 | 1.804 | 72.052 |
| 12 | .810 | 1.688 | 73.740 |
| 13 | .759 | 1.582 | 75.322 |
| 14 | .728 | 1.517 | 76.838 |
| 15 | .716 | 1.492 | 78.330 |
| 16 | .629 | 1.311 | 79.640 |
| 17 | .618 | 1.287 | 80.928 |
| 18 | .555 | 1.157 | 82.085 |
| 19 | .508 | 1.057 | 83.142 |
| 20 | .485 | 1.011 | 84.153 |
| 21 | .453 | .943 | 85.096 |
| 22 | .442 | .922 | 86.018 |
| 23 | .427 | .890 | 86.908 |
| 24 | .397 | .826 | 87.735 |
| 25 | .390 | .812 | 88.547 |
| 26 | .379 | .789 | 89.336 |
| 27 | .366 | .763 | 90.099 |
| 28 | .350 | .730 | 90.829 |
| 29 | .346 | .721 | 91.550 |
| 30 | .322 | .671 | 92.221 |
| 31 | .300 | .624 | 92.845 |
| 32 | .296 | .617 | 93.462 |
| 33 | .284 | .592 | 94.054 |
| 34 | .280 | .583 | 94.637 |
| 35 | .273 | .569 | 95.206 |
| 36 | .262 | .546 | 95.752 |
| 37 | .231 | .481 | 96.232 |
| 38 | .216 | .450 | 96.683 |
| 39 | .209 | .436 | 97.118 |
| 40 | .202 | .421 | 97.539 |
| 41 | .198 | .412 | 97.951 |
| 42 | .180 | .374 | 98.325 |
| 43 | .153 | .319 | 98.644 |
| 44 | .152 | .316 | 98.960 |
| 45 | .141 | .293 | 99.254 |
| 46 | .129 | .268 | 99.522 |
| 47 | .124 | .258 | 99.780 |
| 48 | .106 | .220 | 100.000 |
